# “Building the plane while flying it” Reflections on pandemic preparedness and response; an organisational case study

**DOI:** 10.1186/s12913-023-09874-x

**Published:** 2023-09-01

**Authors:** Karen McKenna, Stéphane Bouchoucha, Bernice Redley, Anastasia Hutchinson

**Affiliations:** 1 Health Services Manager Infection Prevention, Mercy Health, Victoria, Australia; 2 Centre for Quality and Patient Safety Research in the Institute for Health Transformation, School of Nursing and Midwifery, Deakin University Geelong, Victoria, Australia; 3 Centre for Innovation in Infectious Disease and Immunology Research (CIIDIR), Deakin University, Geelong, VIC Australia; 4 Centre for Quality and Patient Safety Research in the Institute for Health Transformation, School of Nursing and Midwifery Geelong, Epworth Health/Deakin University Partnership, Victoria, Australia

**Keywords:** COVID-19, Hospital, Leadership, Pandemic, Operational, Response, Background

## Abstract

**Background:**

The COVID-19 pandemic provided a unique opportunity to learn about acute health organisations experiences implementing a pandemic response plan in real-time. This study was conducted to explore organisational leader’s perspectives and experience activating a COVID-19 pandemic response plan in their health service and the impact of this on service provision, clinicians, and consumers.

**Methods:**

This study was conducted at a large metropolitan health service in Australia that provides acute, subacute, and residential aged care services. Semi-structured interviews were conducted with 12 key participants from the COVID-19 leadership team between November-January 2021/2022. A semi-structured interview guide was developed to explore how the health service developed a clinical governance structure, policy and procedures and experience when operationalising each element within the Hierarchy of Controls Framework. Thematic analysis was used to code data and identify themes. A cross-sectional survey of frontline healthcare workers on the impacts and perceptions of infection control practices during the COVID-19 pandemic, was also completed in 2021 with 559 responses.

**Results:**

Twelve organisational leaders completed the semi-structured interviews. Key themes that emerged were: (1) Building the plane while flying it, (2) A unified communications strategy, (3) Clinicians fear ‘my job is going to kill me’, (4) Personal Protective Equipment (PPE) supply and demand, and (5) Maintaining a workforce. When surveyed, front-line healthcare workers responded positively overall about the health services pandemic response, in terms of communication, access to PPE, education, training, and availability of resources to provide a safe environment.

**Conclusion:**

Health service organisations were required to respond rapidly to meet service needs, including implementing a pandemic plan, developing a command structure and strategies to communicate and address the workforce needs. This study provides important insights for consideration when health service leaders are responding to future pandemics. Future pandemic plans should include detailed guidance for acute and long-term care providers in relation to organisational responsibilities, supply chain logistics and workforce preparation.

The Coronavirus Disease (COVID-19) pandemic has had an unprecedented impact on both the population and health service providers around the world. Understanding of the transmission modes of SARS-CoV-2 has evolved over time, and has at times, been considered controversial with much debate around droplet versus aerosol transmission. In early 2020, the World Health Organization (WHO) identified the transmission of SARs-CoV-2 occurred primarily through droplet and contact transmission [1]. The debate raged on throughout the pandemic and in 2021 the scientific community was reporting on the aerosol transmission of SARs-CoV-2, which was later acknowledged by the WHO [2].

Health service providers were faced with unique challenges influenced by the suite of services they provide, the needs of the population accessing those services, and the dynamic nature of the pandemic that resulted in localised outbreaks and surges in demand for health care resources. Patients, frontline healthcare workers, managers and leaders have all had suddenly and dramatically adapt their services in response to this public health threat while maintaining the safety of staff and services [3].

In January 2020 the Australian federal government COVID-19 pandemic response was implemented to target zero cases, and involved a nation-wide lockdown, travel restrictions on select countries before evolving to all countries, and a two week mandatory quarantine for returning travellers [4]. By May 2020 COVID-19 cases of local transmission and international arrivals were low, with new cases less than 20 per day [4]. However, by June 2020 a second wave of COVID-19 had begun, and localised clusters of cases had appeared throughout Melbourne, Victoria, these clusters were linked to hotel quarantine breaches and were spreading rapidly before public health authorities could contain them [4]. During this second wave of infections the total number of cases more than doubled within a month [5]. Increasing stages of restrictions were imposed on the residents of Victoria to contain the cases, including stay at home orders, mandatory face coverings, curfews, and five kilometres travel and exercise restrictions [5]. Tough border restrictions were also imposed by other States in Australia [4]. By September 2020 a roadmap to ease restrictions was released, and by November 2020 no local transmission of cases was reported [4]. During the second wave of COVID-19 in Victoria the healthcare system faced significant impact, of the 20,000 cases nearly 20% were health care workers, a third of which were nurses and nearly half were aged-care workers [4].

The Australian healthcare system operates through a publicly funded universal healthcare system Medicare, that provides all Australian and permanent residents with access to health and hospital services [6]. Each State and Territory, however, has primary responsibility for providing residents with public health care services within their jurisdiction, including hospitals, public health and emergency management during the pandemic [4]. In March 2020 the Victorian State Government released the COVID 19 pandemic plan for the health sector, in an effort to act decisively and limit the spread of disease [7]. Health services were required to implement a response to the pandemic that executed the government directives, maintain a workforce, and remain operational while minimising the incidence of COVID-19 within their service.

Scientific insights into the nature of the novel SARS-CoV-2 virus developed throughout the early stages of the pandemic and led to the inevitable evolution of evidence about the disease process and viral transmission, and subsequently the government directives were rapidly changing. In response, health services endeavoured to develop and implement a structured emergency response plan that was flexible and dynamic, enabling modification of health service policies, practice guidelines and models of care in real-time, as the pandemic evolved [8].

The aim of this research was to explore the health service organisational response to the COVID-19 pandemic, using a single organisational case study approach. Key factors that were explored were: clinical governance structures, organisational communication, infrastructure requirements, service provision requirements and experiences of front-line health care providers. The pandemic provides a unique opportunity to learn from the strategic response of organisations, by obtaining leadership and clinical staff perspectives on how the organisational response was implemented and the impact of this on service provision, staff members and consumers at a local level. The findings of this study will provide guidance to inform development and implementation of strategies in the event of a future large-scale outbreak of COVID-19 or other pandemics.

## Method

The case for this research is a Melbourne tertiary health service provider. The health service network was selected as the case for this research as it is unique in that it includes a network of hospitals and provides care to the population in both the public and private sectors, and across primary, residential aged care, subacute ambulatory care and acute health service delivery.

During the COVID-19 pandemic, clinical and non-clinical staff and organisational leaders faced considerable challenges continuing to provide care to the communities they service. One hospital within the network faced particular challenges as it provides general acute, subacute and mental services to a population that saw an increase in community transmission and increased cases during the second wave of cases, with 2,248 cases in a population of 270,487 residents [9]. In contrast the specialist nature of another hospital within the network, meant that the health service experienced challenges providing specialist services to women and children during the pandemic as rapid changes in service delivery models needed to be implemented.

### Study design and data collection

A mixed-methods study design was used that included both qualitative interviews with organisational leaders and a cross sectional survey of front-line staff. Semi-structured interviews were conducted with 12 organisational leaders working at the health service during the pandemic. The leaders were selected based on their role within the COVID pandemic response team and were representative of both clinical and operational services across the health service. The organisational structure meant that key leaders had responsibility for programs across all the hospitals within the health service.

A cross-sectional survey was also used to explore clinical and non-clinical healthcare workers’ experiences as part of the organisational response to the COVID-19 pandemic in 2020. Data related to the participants evaluation of the organisational response; staff training and the availability of Personal Protective Equipment (PPE) are reported. The responses relating to side-effects of PPE will be reported separately.

### Recruitment

Purposive sampling was used to obtain a representative sample of organisational leaders from across the health service who had key leadership roles during the pandemic. Leaders were invited to participate in the interviews and were provided with a written informed consent form for study participation. The interviews were conducted over the Zoom® online video platform. Each interview took approximately 30–45 min and was recorded. Inclusion criteria were: Organisational leaders who provided consent for study participation and exclusion criteria were: leaders who were not employed at the health service in 2020 and were therefore not involved in operationalising the COVID-19 pandemic response.

Participants recruited for the cross-sectional survey were front-line clinicians (nurses, midwives, medical and allied health staff) and non-clinical healthcare workers (cleaners, ward-clerks) working across the health service. Exclusion criteria were individuals who did not provide informed consent by completing the online survey, and staff who were not permanent employees.

### Leadership semi-structured interview tool

The semi-structured organisational leadership interview guide was designed to explore how the health service developed a clinical governance structure, policy and procedures and experience when operationalising each element in the Hierarchy of Controls Framework.

There were 12 organisational leaders that participated in the interviews conducted between November and January 2021/2022. The organisational leaders interviewed were; the Executive Director of Nursing and Midwifery and Aged Care Clinical Practice, the Executive Director of Nursing, the Chief Operating Officer, the acting Chief Medical Officer/Program Director Medical Sub-Acute and Palliative Care Program, the Clinical Services Director Perioperative Program, the Program Director Perioperative Program, the Program Director Women’s and Children’s Program, the Program Director Mental Health Program, the Infectious Diseases COVID Consultant, the Group Manager Work Health Safety, the Procurement Supply Manager, the Manager COVID Response. An invitation to participate was extended to the Allied Health program director who was unable to participate due to time constraints.

### Cross-sectional survey tool

At the time of the study, there was no validated survey tool in the literature, so the survey data collection tool was developed with an expert panel of international Infection prevention leaders and was informed by a rapid review of the current literature around issues and side-effects surrounding the widespread use of PPE during the COVID-19 pandemic response internationally and previous outbreaks of respiratory infections [10]. The survey tool was developed through the completion of a series of meetings between the members of the research team, and international experts, including clinical Infection Prevention leads from Singapore General Hospital and the United Kingdom London region, and members of the Australasian College for Infection Prevention and Control [11]. The survey tool was structured in 3 sections, and included a mix of yes/no questions, open ended questions, and a Likert scale with responses ranging from strongly disagree (1) to strongly agree (5), and rating responses on a scale of 1–10 with 1 being very poor and 10 excellent. The survey tool was used to undertake concurrent research at an acute care hospital in Singapore [11]. Demographic data was collected including age, gender, occupation, employment status and work location. The survey questions were based on issues related to PPE that were topical throughout the pandemic.

### Data analysis

The video recordings and field notes of the interviews were transcribed by the researcher who conducted the interviews and checked against the recordings for accuracy. Qualitative thematic analysis was used to identify, analyse, and report the themes identified within the data set. Braun and Clarke’s [12] six phases for data analysis were used as the framework for the analysis. Quantitative content analysis was used to measure the proportion of participants who discussed each theme.

The cross-sectional survey was made accessible to participants via a QR code. The survey responses were analysed using descriptive statistics, including frequencies and percentages. As this was an online survey, we were unable to measure the reach of the survey, however 627 respondents commenced the survey and 559 completed at least one section and were included in the analysis, providing a survey completion rate of 89%. Majority of the respondents were female (83.7%) and aged between 31–45 years (33.6%). Most respondents were Nurses/Midwives (66%), followed by Allied Health (5%). However, 87 respondents (15.6%) did not specify their occupation.

Data triangulation was used to evaluate and validate the responses of the front-line health care workers who completed the cross-sectional survey, and the organisational leader interviews. The cross-sectional survey questions were reviewed, and the questions identified as relevant to the identified themes from the analysis of the leadership interviews were included in this analysis.

### Ethical considerations

Ethics approvals were obtained from the institutional Human Research Ethics Committee (HREC) and Deakin University.

## Results

The organisational leaders were asked to consider the response to the pandemic and the challenges faced by the organisation throughout the pandemic, considering both the preparation and response phases. The themes that emerged from the data were: (1) Building the plane while flying it, (2) A unified communications strategy, (3) Clinicians fear ‘my job is going to kill me’, (4) PPE supply and demand, and (5) Maintaining a workforce.

### Theme 1 Building the plane while flying it

One of the significant themes identified by the organisational leaders was the concept of building the plane while flying it, effectively developing the pandemic response at the same time as executing it. Subthemes identified within this major theme were (1.1) the organisations existing pandemic plan was not fit for purpose, (1.2) a lack of command and control versus egalitarianism, (1.3) a lack of guidance from the health department, (1.3) the volume, velocity, and source of information, (1.4) craft groups implemented independent plans and practices and (1.5) differing tolerance for risk.

#### 1.1 The organisation’s existing pandemic plan was not fit for purpose

There was a consistent observation throughout the participants’ interviews (6, 50%) that the organisations existing pandemic plans was not fit for purpose for a large-scale response to a novel respiratory pathogen. The pandemic plan was noted to be related to pandemic influenza-like events and was considered too abstract with a lack of specific detail (Table 1, Q1 P3). Participants also commented that there was not a direct transference between the influenza pandemic plan and how to respond to an unknown respiratory virus (Table 1, Q1 P3, Q4 P11, Q5 P5). One respondent noted that they were unaware of a pandemic plan existing prior to the pandemic, and the challenges that presented to a large health service in having to develop a pandemic plan while simultaneously implementing it (Table 1, Q2 P2).

**Table 1:** Theme 1, Building the plane while flying it

Themes	Subthemes	Organisational leaders
**1. Building the plane while flying it**	The organisations existing pandemic plan was not fit for purpose	**Q1** “we obviously had a pandemic plan at the beginning of 2020, which was developed for pandemic influenza, but there was an abstractness to that plan, because it had not been developed to specifically combat this pathogen” [….] “the plan had been developed through the lens of this might happen at some point, rather than oh my goodness this is happening” [….] “The other major problem was trying to reconcile whether any of the planning that had been done, from an infection control pandemic management perspective, was going to be relevant and appropriate” (P3)
		**Q2** “At the time WHO was announcing there was a pandemic, there was essentially no pandemic plan, so that’s the starting point”[….] “I remember sitting at a leadership meeting in about March 2020, and you’re asked to develop a pandemic plan” [….]“At that point being advised we were in stage 1, and I said we might as well forget about stage 1 and 2 cause we are already in stage 3 by the time we actually publish a plan” [….] “Various layers of planning were required from a strategic level, running a big health service as part of a big state-wide health system, to what you do at an individual emergency department, ICU, ward level, there was a complete absence of any planning” (P2)
		**Q3** “The original pandemic plan just lacked that granular detail, and some of the discussions we've had over the last two years are just extraordinary in terms of granular details that you just don't think of until you're in the middle of it” (P3)
		**Q4** “I think our preparation for a real contagious virus was well underdone, there hadn’t really been anything since Ebola and not everybody was affected by that” [….] “we based it [the pandemic plan] on sort of flu outbreaks and a few other things that hadn't realistically being reviewed for a while. I don't think people really thought it was a priority until suddenly we were faced with it all, and of course obviously the pandemic sort of snowballed of its own accord” (P11)
		**Q5** “Early on I looked at the pandemic plan that had been written and I just went well that was never gonna cut the mustard was it. We had a very high-level plan of what would happen, and you know that's not what played out, that’s not what happened in a tabletop exercise” (P5)
	Lack of command-and-control verses egalitarianism	**Q6** “So again we had and very much still had today, what’s the hospital down the road doing, are we consistent, we don’t want to be the front runners in case we make a mistake, whereas people working in other jurisdictions the ministry has said you will do this, and that’s what happens. And it creates a different sort of psyche and I think an element of inefficiency which has been significant” (P2)
		**Q7** “it’s easy to run a pandemic you only have to do one thing and that’s run a pandemic, but it’s all the business as business-as-usual stuff which is a lot harder” (P8)
		**Q8** “The history is different too, New South Wales didn't all of a sudden turn on command control, it’s how they operate, it is an aggressive jurisdiction, it’s an aggressive culture up there. And in a pandemic, it absolutely was command and control” [….] “They’ve (NSW) got a centralized model of some of these big pillars, they've got the CEC and ACI, so a lot of the guidelines, a lot of the directions were coming out of the CEC, absolutely. Victoria’s governance in that space isn't command and control. They don’t have the pillars to support a command-and-control approach” [….] “In the beginning everyone was scrambling for procurement and consumables and New South Wales was as well, but it got centralized a lot quicker, and that gave the organization's confidence that that wasn't going to be an ongoing issue” (P8)
		**Q9** “I have never worked in new South Wales, but it sounds like they have a far more streamlined process, they have a far more well developed health system in the sense of different parts talking to each other, and they appear to have managed things at times better than we have in Victoria” [….] “ And I know unhappy people from some of those States who prior to the pandemic bemoaned the dictatorial nature of some people within their health problems and outside of an emergency you could argue that Victoria’s system is better than a more dictatorial top-down approach, but in a crisis you just leaders, you need people running the show so that you have some consistency and you have some stuff in plan and like there's a balance there right, like obviously it gets to dictatorial. I think that de-centralization in Victoria has led to a huge volume of redundant work, I think it has led to a lot of conflict, I think it's led to a lot of inequality in terms of how many people support each aspect and each health service, and I think it's created some problems, but you know we'll see if we learn for next time” (P3)
		**Q10** “it's an interesting thing, whose responsibility is things, is it the health departments, is it the society of infectious diseases, is it the hospitals themselves, you know we've had so much conflict throughout this pandemic between all of those groups as to who should be doing what, it's been difficult to get any kind of coordinated response” (P3)
		**Q11** “I think that command and control and just that focus on making sure that we had one meeting you know, and it could go forever and it's not compulsory, I did say to the team come if you want to come it’s not compulsory and yesterday we had 25 people still on the zoom. You can get the answers, you can ask the questions and then we can all move on with a decision” (P8)
		**Q12** “So I just make a decision and you know I’ve got to back myself, and I get advice if I don’t know things, I’m not that precious that I can ask the question. But to be able to get the information, make the decision and move on, so everyone else can move on, and to have confidence that we know that I am with them if it all turns to shit you know” (P8)
	Lack of guidance from the health department	**Q13** “The Victorian information was probably the better of all the States and the Commonwealth”[….] “the Commonwealth information and the Victorian information would often conflict, so we would try to look at what was the best information we thought we had at the time” (P1)
		**Q14** “some of what we were being given is very difficult to operationalise, and probably at times was given to us in a way that almost makes it impossible to operationalise” [….] “it's such a challenge, getting that balance right between giving people instructions with enough detail that they can follow them, but not so little that they can follow them without necessarily knowing how they can operationalize them. At times there appeared to be some internal contradictions” [….]** “**They were giving people instructions that lack some of that granular detail, but then it's very difficult to operationalize cause you are trying to think what do you actually want me to do” (P3)
		**Q15** “The information coming from the Department was sporadic, it was haphazard at times, it was unclear, It was duplicating, I found it really reactive and not proactive” (P11)
		**Q16** “In the first several months there was a plethora of documentation and I guess one of the principles that the health leadership group had to stick with was, if there is a document from the department we should stick with it because we have got a defendable position, but the absence of documentation was a real challenge and the void was being filled by people in management roles and by clinicians however they saw fit” [….] “We still have elements now, well beyond those sorts of days where different elements of government and the department issue documentation which sometimes are not consistent with each other, or in fact in straight conflict with each other in terms of some of the detail that needs to be applied in the workplace” (P2)
		**Q17** “The department was not on the front foot about communicating things in a pragmatic and clear way to those they send out directions of what to do” [….] “The department information would make no sense when you try to translate it into an operational environment, they send out all this kind of principle-based information without thinking about who's going to be doing the work” (P9)
		**Q18** “We were given a set of principles and we were going away to actually interpret what we thought those principles were” [….]“I think in terms of guidance we would have preferred perhaps more explicit guidance” (P10)
		**Q19** “I know a lot of staff got very confused at times with what are they asking us to do. And if the instruction was relatively clear, it was well how's that going to impact our health service, because you know each health service can I guess interpret as you like, like so does that mean we have to do A or do we have to do B” (P11)
	Volume, velocity, and source of information	**Q20** “And depending on where we were in the process, the advice was changing if not weekly, daily, on what you could and what you couldn't do, what was on what was off” (P4)
		**Q21** “In terms of the actual advice and the guidance itself, the volume of information coming out was very chaotic and it was hard to keep across, and to then be able to disseminate to my team in a timely manner” [….] “there were days where you would get up to 2, 3, maybe even 4 separate bulletins come out from government that all could be about the same thing and providing up to date different advice, so the one in the morning would have different advice by the evening” (P12)
		**Q22** “I also understand that obviously there was a velocity to this, the changes that I think was always going to be challenging and difficult to go through” [….] “The timing of a lot of that communication was less to be desired. It was quite often a Friday afternoon at 5 pm and there were circulars that were provided to us, which we had to essentially try to communicate to the rest of the health service before packing up to go home on the weekend” (P10)
		**Q23** “And just the level of information coming out was really high volume and trying to filter that to what was relevant and what people could actually absorb in one hit was really challenging” [….] “It was such a new disease, they really didn't understand it, nobody in the world really did, so we were really just sort of rolling with whatever information we were provided, and a lot of the information that came from the Department was really late on a Friday, with directions of what health services had to respond to, or implement and we were constantly doing it” (P11)
		**Q24** “At that stage by then we actually we counted that we had 13 different agencies come into the home over that period of time and would tell the staff something different” (P1)
		**Q25** “One of the major challenges was around information coming through thick and fast from a lot of different avenues, including the media, we had to develop fairly quickly a communication structure that was going to mean that we could get managers and leaders the information that they needed, in order to perform their roles in the safest possible way (P9)
		**Q26** “Initially a huge variety of information was being distributed by multiple people across the organization, so people were distributing information as a come out of Europe, from other hospitals, from professional bodies, and it wasn't the same information, there were a lot of discrepancies in it. It was creating a panic, and that was the first sense of we didn't have any control over the information going around the entire organization, and the volume of it” (P1)
	Craft groups implemented independent plans and practices	**Q27** “The absence of documentation was a real challenge, and the void was being filled by people in management roles and by clinicians however they saw fit” [….] “Working with a workforce what we found as one of the major challenges was the various craft groups would come up with their own professional document from just about anywhere in the world to answer a clinical issue that had arisen in relation to what sort of PPE should we be using, under what sort of circumstances” (P2)
		**Q28** “We then had to explain to us why we were making some of the decisions we were, trying to provide that reassurance that that the information we were providing was based on best practice, based on trying to balance demand was in agreeance with guidance coming out of the department, so that that period was very challenging because obviously from an individual staff member perspective, there was a lot of differences of opinion from individual staff as to why some of the decisions were being made” (P3)
		**Q29 “**Certain craft groups were very active in developing their own guidelines, there was obviously positive aspects to that, the people were looking at their own workflows and situations and trying to develop appropriate guidelines, but at the same time that created a lot of conflict because certain groups were mandating certain things and others weren't, and there was a lot of conflict there. We had one situation where a craft group was manufacturing their own PPE and we had to try and deal with the concept of you can't do that, like you know this is not safe” (P3)
		**Q30** “And there were concerns across the different craft groups, say anaesthetics for example who had undertaken measures which I think were probably decisions made by individual departments without the right governance” [….] “And I think professional craft groups felt that some groups were perhaps more equal than others. So again there was that tension as well, and it was difficult because even though the department had a particular view about that, it wasn't necessarily accepted that that was the correct view” [….] “You know if it was good enough for the anaesthetists, it was good enough for the intensivists, it was good enough for the emergency physicians” (P10)
		**Q31 “T**here was some challenges in terms of managing individual personalities, as well as craft groups who again had a view that perhaps they should have been vaccinated earlier, or they should have been actually in that first group” (P10)
		**Q32** “People would come up and I could hear them chatting outside my office, and we're going to do this we're going to do that, I kept saying we’re not doing anything that's not approved through the governance committee. I mean, I can make decisions on the run, but I’m not doing it” (P8)
		**Q33** “All the maternity tertiary hospitals put together an expert working group and got together every week and really all trying to make decisions at the same time, so we were trying to really move together as a group, which was not that easy to do. Like it sounds like it would be something that we would make a decision and then all go with it, but then we also had our executives that we have to go back to” (P6)
		**Q34 “**I was advocating ahead of the rest of the state to say actually I think my team should probably be wearing a higher level of PPE than what the Health Service was recommending. We did manage to convince them of those needs on each occasion when we needed it, sometimes there was tension saying all you know that's going to increase the usage, and I would say well that increases the usage at least I've got staff” (P9)
	Tolerance for risk	**Q35** “As an infectious diseases physician I not infrequently have to treat people who have diseases that I could contract, so you cognitively get an opportunity to get past that, whereas for a lot of doctors it's very uncommon for them to ever come into direct contact with a patient who has a medical condition that they might contract and might actually cause them some harm” [….] “When I started as an infectious diseases registrar I started at a point where we had a measles outbreak, and so I am vaccinated against measles but it's not a disease you are used to seeing, I had to go into rooms with a highly infectious pathogen. And I know for me it was a process, particularly with little kids at home, it was a process of being like okay, I'm doing this, the PPE works, I follow the rules and I follow the instructions I'm going to be okay. And then I was okay. Then COVID comes along and you're already used to that, follow the instructions do the process right, wash your hands, blah blah blah and you'll be fine, I think for a lot of other staff that wasn't something they necessarily really confronted a number of times before, and obviously your mind wanders in those situations and we saw a lot of that, what about my wife, what about my kids, what about my whatever whatever, and that side of things certainly made things very difficult” (P3)
		**Q36** “I will note as a clinician, that having been around long enough and grew up effectively as an early consultant in the HIV era, I have long held the view that health care might kill me, and maybe its just me but there were some contextual issues around how you’ve developed your philosophy around what you do as a health care provider. I think people who haven’t had that previous exposure have grown up with a degree of ignorance of the fact that health care is potentially a dangerous environment, not just a ticket to earning big dollars speaking from a medical staff point of view. And as we have seen things like mandatory competencies for hand hygiene, or donning and doffing, use of PPE, some of the anaesthetic group kind of would be the last group, absolutely refused to wear masks, yet I’ve been in meetings 18 months ago where I have been personally threatened for potentially killing anaesthetists by not providing them with masks” (P2)
		**Q37** “These are the things I've noticed the most, is how much different craft groups tolerance for uncertainty and their tolerance for risk are totally different. You feel like you've got your head around something and then you'll go and try and deal with another craft group and they’ll be totally not on board with it because their tolerance of risk of their tolerance and uncertainty is totally different and that's been a huge challenge” [….] “At the medical level ED and anaesthetics are the two most opposite extremes, anaesthetists are very risk averse and don't like uncertainty, whereas emergency doctors tend to be far more just give us a bit of instruction and we'll just run with it, because you know our lives are fairly chaotic and that's how it is. And it's been a really interesting aspect of all of this” (P3)
		**Q38** “Many people around us quoted whoever it was within the WHO in response to pandemics, or response to disaster situations, where you have to be agile, you have to actually come up with decisions quickly rather than go through 15 committees and have bureaucracies” [….] “At exactly the same time we are going through hospital accreditation which is almost the antithesis of this, where you have every nut and bolt in place and you have every clinical guideline reviewed and up to date and sits on an electronic register, and yet here we were creating guidelines that were just literally being churned out of, in some cases, one individual persons notebook, without going through those checks and balances that we sort of take for granted, so that was quite challenging” [….] “We were used to having that sense of security around us in terms of, they can come up with a plan, consult with people, go through a committee if it needs a bit of fine tuning does it really matter. But now we are actually a very risk-taking environment which requires us to be there because we have to do things quickly, and so a lot for people in management felt very uncomfortable about that” (P2)
		**Q39** “So I think back to the very beginning, and it was just chaotic. We were getting information coming left, right and centre, and there was an enormous pressure on us as a team to make sure that we did everything right, whatever that was, and the overwhelming responsibility of not being the ones to let the cat out the bag” (P6)
		**Q40** “And waiting to hear from say other health services, what they were doing, an example is of what tier PPE people were wearing and the overlay of emotion that was associated with that, the changes that were happening with our neighbouring health services that had undertaken an escalation of PPE when that wasn't very clear that was what the department wanted, but it became essentially customer practice because it was driven by the concerns of the staff” (P10)
		**Q41** “There had been no training around incident responses at the organisation for a long time, I don't know when the last one was” [….] “the management team in health services didn't understand how incident command works under a crisis, because there's a decision made and its implemented. The consultation period and the discussion and debate can’t happen when you're dealing with a crisis, and the health services team found it really, really difficult to change that approach. So we get considerable complaints from staff that were not getting rapid information because it was being blocked by inability to make a decision and getting consensus. The whole thing around incident command is make a decision with what information you have at the time, it's not necessarily the right decision, and it doesn't mean that that process won’t change as circumstances change, but you get on with it” (P1)

#### 1.2 Lack of command and control versus egalitarianism

The perceived lack of a centralised command and control model within the Victorian Department of Health during the pandemic was identified by five (42%) participants as a barrier to implementing an efficient response. The participants noted the difference in the Victorian response compared to other States in Australia where a command and control model was in place (Table 1, Q8 P8, Q9 P3). Three (25%) participants identified the information received from the Victorian Department of Health response came in the form of guidelines requiring interpretation, and the subsequent lack of coordinated responses between health services leading to redundant work, inequality, and conflict between health services (Table 1, Q6 P2, Q10 P3).

#### 1.3 Lack of guidance from the health department

There were consistent responses from 50% (6) of the participants that the guidance and information from the Health Department to the health service was a set of principles rather than specific directives, requiring interpretation and translation into usable resources for implementation (Table 1, Q14 P3, Q17 P9, Q18, P10). It was also identified that the guidance differed between States and the Federal health departments, creating challenges for organisations providing services, (such as residential aged care facilities), in more than one state (Table 1, Q13 P1).

#### 1.4 Volume, velocity, and sources of information

There was a consistent perception from 50% (6) participants that the information, advice, and guidelines were changing so frequently that there were significant challenges in controlling the dissemination of information throughout the organisation, resulting in confusion within the workforce (Table 1, Q20 P4, Q22 P10, Q23 P11). The need for a clearly structured communication plan was identified by one participant who noted that at the beginning of the pandemic when information was slow to come from the Department of Health, the wide distribution of information by the workforce from varied sources including the media, different professional bodies, and observations from the earlier European response, was creating a sense of panic (Table 1, Q26 P1).

#### 1.5 Craft groups implemented independent plans and practices

Five (42%) participants identified the challenges of working with different craft groups, who would use the lack of clear directives as an opportunity to produce their own documents in response to clinical issues (Table 1, Q27 P2, Q29 P3). While there were positive elements to this practice, in that staff were actively looking at their own workflows and planning, it also created conflict between different craft groups and at times a lack of governance, (Table 1, Q30 P10).

#### 1.6 Differing tolerance for risk

A differing tolerance for risk was identified as a subtheme across individuals and craft groups by 6 (50%) participants. Two of the participants identified within their own clinical practice that they had accepted that they would be exposed to infectious diseases as clinicians and had developed a philosophy around their practice to address that risk (Table 1, Q35 P3, Q36 P2).

One of the participants identified different craft groups had differences in tolerance for risk, and this was a barrier to streamlining the implementation of the organisations pandemic response (Table 1 Q37 P3). Challenges appeared to stem from a lack of understanding by clinicians of the emergency pandemic response (Table 1, Q41 P1), and the need to move quickly with decisions therefore bypassing the consultation process normally required within the governance structure (Table 1, Q38 P2, Q41 P1).

### Theme 2 A unified communications strategy

The second major theme identified was the need for a clear and consistent communication strategy throughout the organisation. Sub themes that were identified within this theme were (2.1) consistent communication within the leadership group, (2.2) discovering the need for a structured communications strategy, (2.3) changing goalposts.

#### 2.1 Consistent communication within the leadership group

Five (42%) of the interviewed participants identified the cohesive communication between the leadership group, and the frequent meeting of the group as being an important element to a successful and unified response. As well as ensuring all the right people were in the same room getting the same message, disseminating this throughout the organisation to make sure everyone was on the same page (Table 2, Q38 P2, Q41 P1).

**Table 2:** Theme 2 A unified communication strategy

Themes	Subthemes	Organisational leaders
**2. A unified communication strategy**	Consistent communication within the leadership group	**Q1** “Cohesion at the management level and regular meetings, where all of the senior leadership team are in the room at the same time and are sharing information has been very important” (P2)
		**Q2** “The communication within the leadership group was quite good, the ability to rely on our infectious disease consultant and his expertise and availability was really quite a strong point” (P11)
		**Q3 “**We tried to have a unified response so if we came up with something it didn't just mean going back to that individual going oh here's what you do, it actually meant we need to think about this from a broader perspective, here’s how we should implement it across the organisation” (P4)
		**Q4** “I met with the NUMS, and I held a couple of staff forums, and you know I met with the educators, I met with the grads, and you know started to try and slice an organisation so every was everyone was getting a consistent message, I think it's important (P8)
	Discovering the need for a structured comms strategy	**Q5** “In the beginning we did not know what was happening from an organisational level, you'd hear things were coming, but it's like so is anyone actually doing anything about that or do we just do it ourselves, well I can't sit here and not do anything because I’ve got a whole staff group that I need to take care of” (P5)
		**Q6** “Communications has got to be a key important strategy, and It's got to be timely and responsive and to the point for people so that they know” (P5)
		**Q7** “Comms was centrally driven, we had visual posters that we changed the message and changed the colours, so we were constantly refreshing the message” (P1)
		**Q8** “One of the challenges was we tried different forms of communication, people said they weren't getting enough, people said we're going too much” [….] We had visual posters that we changed the message and changed the colours, because after a while people stopped looking at them or couldn't see them” (P1)
		**Q9** “Situation reports and communications are good, even if you can go back to those and pull them out again and refresh them and send them back out to staff” (P4)
		**Q10** “Rather than me as a leader directly telling all the individual anaesthetists what to do, I’ve got a head of department using their ways of communication, so leave enough responsibility with people who are in positions of authority to use their usual ways of communicating, as well as what we needed to put out as a whole of health service on top of that” [….] “And not relying on single channels of communications, so if there is information that needs to go out, it goes through nurse manager groups, the CMO office, CEO communiques, sits on workplace by Facebook, in emails, so multiple channels of communication” (P2)
		**Q11** “we had to develop fairly quickly a communication structure, within the program that meant we could get managers and leaders the information that they needed in order to perform their roles in the safest possible way” [….] “We had daily forums with leaders, so that was our verbal communication structure, but you need written communication in order to support the verbal communication” (P9)
		**Q12** “I think the creation of completely novel subcommittees which have dealt with things like vaccination, rapid antigen testing, the PPE subcommittee which went on to respiratory protection, those structures have been absolutely critical in terms of sharing of information” (P2)
		**Q13** “Within 6 to 12 months, we had a lot of good subgroups, the frequency of meetings was good, the information was certainly better, I just don't think it was brilliant getting down to the staff” (P11)
		**Q14** “Communication was key, and you can tell people and tell people and tell people, and you will still find that not everyone checks their emails, and we have such a reliance on this email communication” [….]“But you're talking to a group of staff who are really busy, overworked, have a lot on their plate, and every time they turn around there's something new that they have either got to do, find, tell, get” (P4)
		**Q15** “We needed a dedicated COVID response manager to try and create some consistency of approach, to try to strengthen the comms and get visualization of what needed to happen out to the teams, and know where the resources were sitting. If you look at other bigger organisations and they got a team managing their COVID response” [….] “And we were under resourced and probably still are under resourced for what we're managing to be honest” [….] “But the COVID response, for what was needed, that just fell back on the local areas to magic up what needed to happen or to work out what to do. I could see we need to do this, we need communication in our local area because people are panicking if you don't give them anything” (P5)
		**Q16** “There were resources sitting in local areas everywhere, nothing was aligned, there were lots of local bits and pieces” (P5)
		**Q17** “I also think that the timeliness of communication coming out from the organisation at the highest levels from a Chief Executive space could have come out more rapidly” [….] “I found myself doing a lot of interim information to fill the gap in those early stages of communications that were coming out centrally, because staff needed to know, and it was a bit repetitious, but they felt comforted by the fact that we would send something out every Friday afternoon, so we know what to focus our energies on” (P9)
		**Q18** “And I think we could always improve our communication, I don't think we really put it out really regularly, I don't know if it was in the right format that everybody could take good points away from” (P11)
		**Q19** “There was a lot of information coming from different sources, even in email and staff feedback, it was what do we look at, I don't know what to look at, it's too much to look at, so I think in the programs we worked hard to try to condense it” [….] “And I make sure that every communication that’s sent as relates to COVID, I embed that in that meeting so everybody knows there is one place you can go to for everything in the week, so funnelling information for staff, I think we could have done better as a leadership group too” (9)
		**Q20** “I think the communication within the leadership team was good, maybe not so much down to the staff on the floor because there was just such a volume, we had to really filter and pick and choose a little bit” (P11)
		**Q21** “I think the communications came eventually, but they were difficult to work out from a manager perspective, they were really long, the important information was hidden in little paragraphs on page number 6 in paragraph number 4 or something, you know the key things” (P5)
		**Q22** “I tend not to accept in this day and age that people don't answer their emails if you can book a holiday online, that seems to come up you know that I don't access my emails, I tend to not put much weight into that these days” (P8)
		**Q23** “The ability to zoom has raised a different audience as opposed to try and pull everybody in from the wards into a big auditorium, this is a lot more agile, a lot more nimble of a communication style and we really should continue to do it” (P8)
	Changing goalposts	**Q24** “We were getting changes from the Department sometimes twice a day. So the rapid process of communicating changes in the organisation for that sort of response was incredibly challenging” (P1)
		**Q25** “It was a changing feast all the time and to try and get that communication out there to people” [….] “The staff were actually getting really frustrated with us because we were changing things, but we were trying to be agile and trying to get the right balance” (P6)
		**Q26** “There was a lot of trying to get that messaging out and around and using all those communication platforms to do that, and then also checking to make sure staff were doing it, and trying to find those resistors in the groups, and then find your adopters and spread the word” (P5)
		**Q27** “It was a bit of a no-win situation, trying to provide consistency, which was difficult because things would change, the goalposts are changing so quickly” [….] “Then we'd put something out, and then something else would come out, and there was conflict a little bit in everyone would go into ahhhhhh” (P5)

#### 2.2 Discovering the need for a structured communications strategy

The participants (6, 50%) identified the challenges in implementing a unified communications strategy across a large health service, with both positive and negative outcomes identified. Positive outcomes of the communication response were having a consistent strategy that was centrally driven and used multiple platforms to reach different audiences, including the use of electronic meeting platforms, and posters and messaging that could be refreshed and updated regularly (Table 2, Q5 P5, Q7 P1, Q9 P4, Q23, P8). Negative outcomes of the communication response included the challenges associated with identifying the frequency of communicating with staff and finding the balance between providing the workforce with enough information to provide guidance but not too much that it was overwhelming and confusing (Table 2, Q8 P1, Q14 P4).

Timeliness of communications to staff within the organisation was identified as an important aspect of the communication strategy, the frequency of this was contested with some leaders reporting positive outcomes and some reporting negative outcomes. Four (33%) participants identified that timely and responsive communications were an important strategy, but there were delays and issues with getting this information down to the clinical workforce (Table 2, Q13 P11, Q17 P9). And the delays in communications down to the workforce led to the local areas developing their own messaging, which resulted in inconsistencies in messaging, and resources sitting in multiple places (Table 2, Q5 P5, Q16 P5, Q17 P9).

#### 2.3 Changing goalposts

There was consensus among five (42%) of the participants that the frequency of changes to the guidelines created barriers in establishing a consistent unified communications strategy. Communicating the frequent changes in the guidelines across a large organisation rapidly, was difficult and created frustration within the workforce (Table 2 Q24 P6, Q25 P6, Q27 P5).

##### Cross-sectional survey response

When surveyed, the front-line healthcare workers responded positively about the communication from the health service during the pandemic. The respondents were asked to rate the communication from the health service to frontline staff about changes to protocols and procedures, on a scale of 1 to 10, with 1 indicating they were not at all confident and 10 extremely confident, 427 staff responded with a mean of 7 (SD 2.35). Staff were also asked to rate the overall organisational response to the COVID-19 pandemic, 419 staff responses, with a mean of 6.99 (SD 2.38). Indicating that the workforce was reasonably confident in the health services response to the pandemic, with some room for improvement.

### Theme 3 Clinicians fear ‘my job is going to kill me’

The third major theme identified was around the fear of the unknown, and the subsequent challenges the leadership group experienced in implementing the pandemic response across a large workforce. Sub themes identified within this major theme were (3.1) fear of the unknown, (3.2) getting staff into the mindset of a pandemic, (3.3) infection prevention confusion, (3.4) developing novel PPE programs, and (3.5) adapting to change; implementing PPE processes within the workforce.

#### 3.1 Fear of the unknown

The subtheme of fear was raised by five (42%) of the interviewed participants. Fear was reported as being multifactorial and included staff being scared to attend work, the impact of fear on human behaviour, and the impact of fear on patient care.

The participants reported that the fear of the unknown experienced by the workforce, included the risk for their own personal safety and of acquiring an infectious disease, as well as the risks associated with taking the disease home to their family (Table 3, Q2 P4, Q4 P2). The consequences of this impacted patient care with staff being afraid to attend work, and afraid to enter dedicated COVID-19 zones (Table 3, Q7 P1).

**Table 3:** Theme 3 Clinicians fear ‘my job is going to kill me’

Themes	Subthemes	Organisational leaders
**3. Clinicians fear ‘my job is going to kill me’**	Fear of the unknown	**Q1** “There was a lot of political challenges, news, fear from some of the staff around could you come to work, should you not come to work” [….]“So let's also remember the team of people with the supply want to stock the shelves and then our cleaning and housekeeping staff getting rid of this stuff, and going well hang on a minute am I going to get infected with me handling all of these infectious waste bags, so we just had it from end to end, so yeah the donning the doffing, but the getting rid of the stuff at the end of the day” (P4)
		**Q2** “Staff were just nervous and scared, and really early on you couldn't keep a bottle of hand sanitizer on a table because people would steal it. I want to take you back to that time where gloves and gowns and masks were walking out the door in insane quantities, as well as toilet paper as we all know” (P4)
		**Q3** “We had situations where a certain craft group was manufacturing their own PPE and we had to try and deal with that concept of you can't do that, this is not safe” (P3)
		**Q4** “Front line staff were initially very scared, they were afraid of the unknown, their own personal safety, personal safety for their colleagues and their family members as well as the unfamiliarity of things happening quickly around them” (P2)
		**Q5** “The Doctors fear and the disinformation was shared and spread through the nursing staff, because it was coming from that source it was quite interesting, almost like the Doctors lost the ability to think” [….] “It’s like a Bambi in the spotlight, so frightened about what might happen that they couldn't seem to work through it” (P1)
		**Q6** “the issue for me was the level of fear in the medical staff, and again people deciding things based on that fear, rather than going to people with more expertise” [….] “so there's certain groups that were more panicked than others, the anaesthetists at Heidelberg certainly” (P1)
		**Q7** “It also raised a whole heap of things such as the residents in the hot zones weren’t getting basic care, people were too frightened to go in there. People that were furloughed were too frightened to come back to work, so all those things started to come up” (P1)
		**Q8** “Some people were very paranoid, and there was some not nice behaviours around dealing with people that didn't have the right PPE on” (P11)
		**Q9** “If there's a lot of anxiety everywhere, your job is to recognize if you have any for yourself and find ways of managing it, then you're more able to assist others to recognize that, so I supported my leaders to try and manage their own anxieties about the world, the life, the uncertainty of things, so that they could assist people reporting to them, and then so on and so forth. They then managers had to do it with team leaders, and team leaders with clinicians and clinicians with clients” (P9)
		**Q10** “Trying to get people in a place where they felt like they were going to be okay, and to be at work and to still provide care was a big challenge” (P5)
	Getting staff into the mindset of the pandemic	**Q11** “You need to get everyone to watch the movies like Outbreak and Contagion and get your mindset happening when people are thinking about the donning and doffing processes (P4)
		**Q12** “We were telling staff to do this but they didn't always understand why, they knew it was to protect them, but they didn't understand sometimes what the PPE was actually doing, that it was protecting both parties, the patients and the staff” [….] “Getting staff to understand the why, like you can tell them to go and have a cup of tea, but understanding that you do it to stay hydrated is a whole different kettle of fish” (P11)
		**Q13** “So massively steep learning curve to get people familiar with it, comfortable with it, then using it appropriately, using the language appropriately, setting up the donning and doffing stations, so that was a big challenge for mental health that we didn't have to deal with before, whereas in the physical health space I think there was a greater level of comfort and familiarity with that” [….] “It was a steep learning curve for mental health, if you think about what happens in a that environment, unless you're caring for somebody with TB or similar, people were not really familiar with using PPE” (P9)
		**Q14** “Human behaviour was the problem again, people have forgotten about wearing masks and making sure that they didn't take them off, forgotten about social distancing and all the key elements” (P1)
	Infection prevention confusion	**Q15** “It was a challenge to actually be able to explain to people what the difference of PPE was, like the different levels of masks” [….] “After a little while we actually realized there were little cohorts in areas that needed some focus on, so they created the PPE oversight group” [….] “Here’s some people who were very qualified and experienced staff, and we have to try and explain to them what proper eyewear means and why you are wearing it, and not to pull your mask off every five minutes, and getting people to be fit tested, nobody's heard the term fit tested before, and didn’t know why” (P11)
		**Q16** “The midwifery group haven't had a lot of experience working in a general hospital before so the thought of actually looking after people that were highly infectious was very foreign and very scary to that group” [….] “Things that you would normally think people would know and take for granted, people didn't know. Fancy that, doctors didn't know how to get a gown on and off safely” (P5)
		**Q17** “Once we had actually figured out what are the differences between a surgical mask and a N95 mask, because nobody really heard the terms much before, depending on which area you worked at a lot of the ward staff had no clue what a N95 was” [….] “The ability to don and doff, some people hadn’t even heard the term donning and doffing before, so getting all of that embedded into staff was a real challenge” (P11)
		**Q18** “You're trying to reassure people that you have their best interests at heart, that you're doing everything you can within guidelines, there's not a lot of high quality evidence for PPE usage so it was very difficult to go to the literature and say here's the literature that shows X, Y, Z, percentages reduction in transmission, based on using this PPE verses that PPE, so it was very challenging” (P3)
		**Q19** “To mask or not to mask, so very early on in the pandemic there was a lot of debate, I wanted to wear a mask and the organisations position at the time was that a healthy person does not need to wear a mask” (P4)
		**Q20** “Aerosol generating procedures specifically generated huge fear and anxiety from the very beginning of the pandemic. And then there was this whole creep from aerosol generating procedures that are to broadening that definition to all sorts of other things, I remember back then oh we're drilling into people's bones and the drill is going to be creating aerosols, or we're doing this and this is going to be.. it just got out of hand. I think there's an arbitrariness to some of this, like someone has to draw a line somewhere and say this is what droplet spread is, this is what airborne spread is, when obviously we know that all these things come on a continuum in terms of how infectious something is by start a whole range of factors” [….] “I think people were getting very hung up on a fixed definition, which is important but at the same time we knew that some of the paradigm about droplet spread was probably not true, and that this pathogen was probably more infectious than a traditional droplet pathogen, but also not as infectious as a true airborne pathogen” [….] “You're trying to have these discussions with people and quite often it would just boil down to droplet or airborne, and well it's a bit more complicated than that, like there's an arbitrariness to those definitions. Because you know you have to eventually draw lines and say this is this, and that's this, but the real world is obviously so much more difficult than that” (P3)
		**Q21** “Even some definitions from anaesthetists around what is an aerosol generating procedure, the epidemiology of this and is it droplet or airborne, I mean there was some confusion around the actual disease itself. There was some confusion around the actual disease itself, and some of the precautions or flow on effects of how we managed the worksite not only in PPE but things like how we disposed of people within the workplace” [….] “It is probably more like the nursing staff in recovery taking a LMA out that is actually potentially at more risk than an anaesthetist under most sort of circumstances in reality. And then the whole conversation about whether someone huffing and panting in labour in the birth suite is an aerosol generating behaviour as opposed to procedure, all sort of had to be worked through” (P2)
		**Q22** “There was news that staff had heard locally or internationally by colleges about decisions about how much PPE had to be worn, whether you need to wear PAPR suits because your exposed to aerosols, so there was a heightened anxiety and angst really” (P10)
	Developing novel PPE programs (education, fit testing)	**Q23** “Our PPE education program got much, much bigger, so from an upskilling of staff, wearing gowns, gloves and masks, was traditionally seen as infection control only, it's only there to prevent you from contaminating the patient, now it includes staff safety too” [….] “There was a shift in that focus to say PPE stops infections and it protects you, this is why we need you to wear it, lots more education, people now need to refresh their PPE knowledge every six months” (P4)
		**Q24** “After a little while we actually realized there were little cohorts that needed some focus, so they created the PPE oversight group” (P11)
		**Q25** “I think we got videos from Tasmania, not that I have any problems with Tasmania, but why can’t we do that, and then someone said I work at Monash here’s their version” (P2)
		**Q26** “The SA department of health just churns out this is what you’re going to do, doesn’t matter which department you’re in, and they just do it. They had all of their staff donning and doffing with the Doh SA guideline, with the right PPE in about march of 2020” (P2)
		**Q27** “All the systems were put in place, we did donning and doffing practice sessions, the online component of that was developed, and it wasn't there instantly, there was something, but not currently what we've got” (P1)
		**Q28** “we are operationalizing that [education] and moving that into the role of the learning business team, who are all clinical and they will become the experts at assisting with the infection prevention leads within each home” (P1)
		**Q29** “we have our own learning and development unit within the mental health program and we basically deployed them to that task really quickly so that they would become experts. And they could become spotters and they could use the train the trainer model so they learnt all that they needed to off the experts, then they come and translated that into our environment” (P9)
		**Q30** “let's bring in a program where we need to fit test 3000 healthcare workers, let's do that, that sounds great, I don't have the equipment, the trained staff, clear processes, clear guidelines. I've got this great document from the Department of Health which tells me this is what you should do, but we're also operating in the healthcare industry that's probably never even seen a P2 mask before. And record it, and make the data available, make sure people know which mask they need to wear, I could go on” (P4)
		**Q31** “Werribee had been very exposed to COVID, especially in their high-risk areas so they were all on to it (fit testing), they wanted to be safe, they didn't want to take it home to their family, they were much more into let's go and get fit tested. The managers of those COVID areas were like you go and get it organized and get fit tested” (P5)
	Adapting to change; implementing PPE processes within the workforce	**Q32** “So trying to keep up with what was required from PPE and trying to get everybody to understand it was a challenge” [….] “There was a lot of confusion and too many changes, so you had all our different levels of PPE for COVID peak, green and orange, and then suddenly you're in black, and that's when the Department had put out a message saying this is the level of PPE but you need to have a look at that as an individual health service as well” [….] “And what level of PPE we were having for what scenario, what circumstance and what ward. And sometimes we would have three or four different PPE levels within the one site, depending on what that level of risk that was associated was” [….] “it was like well we've got two very different hospitals, two different cohorts of patients, so one hospital was at a lower level of PPE, one was at the other, and then you had staff going between sites, so then they got confused with what level they were wearing in what area” (P11)
		**Q33** “Can I just say people lose their minds pretty much every time there's a small change” (P11)
		**Q34** “We've come so far, I remember back when we first had to wear masks, like we had to put a mask on you, the sky was falling in, this is just a normal mask and everyone whinged about wearing masks” [….] “And the transition to ear loop masks, and there was a thing and all of this hoo haa that went on every day about masks. Were they worn right and wet and how long did you wear them for, and this that and the other, and now everyone's wearing N95s all day and you’re not hearing a peep. And we're not hearing anything about you know my ears hurt and this that and the other, and it’s just like the transformation has been phenomenal, so I think we've come a long way in the PPE world” [….] “Once upon a time that would have been a six week lead up. You know you wear a mask and this is how you do it, this is how you put it on, this is how you take it off, and this is what you do or what you don't do, whereas it was rapid change and frequent change of PPE or all sorts of things” (P5)
	Implementing processes with PPE	**Q35** “It was just repetition and support about that repetition, so we were very mindful from the beginning to try and bring in processes that were very kind of clear and reproducible. A lot of it centred around we used a lot of visual cues, so it was quite easy, staff didn’t have a lot to remember it was the visual cues were there, and then staff would just follow the process. And then obviously they got to do that over and over again during wave two, and then when wave three came along they were really ready and felt supported in that, and we could just re-action everything. As opposed to one site where we did a lot of that stuff leading into the second wave, and didn't end up using a lot of it because we really didn't have that many patients, and then when it came time to do it again for the third wave it was a lot more challenging because a lot of the staff hadn't had that opportunity to practice, to get over that mental hurdle of okay I'm dealing with COVID patients if I follow the rules and the processes I'm going to be okay so that was probably the main difference” (P3)
		**Q36** “It was hard work for a lot of the clinical staff, and from the management perspective, it was really hard and you had to try to lead by example and have all the right PPE on yourself. To be able to try and encourage everybody else and show them how to do it, so there's a lot of training and education required on the use of PPE, once we actually had the stocks of it” (P11)
		**Q37** “Yeah it was frankly an issue around human behaviour, and it's still a challenge for us” (P1)
		**Q38** “In the early days staff couldn't wait to get into the N95’s and the gowns, like everyone was really relieved because they kept thinking oh why aren’t you putting us in PPE why aren’t you protecting us. And then really that first wave when we did put them in N95s and then they realized how unpleasant it actually was working in those conditions, that this time around they have been more reluctant” (P6)
		**Q39** “And then the other side of it was actually getting people to wear it. It (PPE) was uncomfortable, like a lot of the reasons people didn't like it was it was uncomfortable, unless you worked in theatres, some areas people weren't used to wearing masks all day” (P11)
		**Q40** “I know that you put a P2 mask on and someone says you're going to wear that for your 8, 10 or 12 h shift, exhausting” [….] “Wearing a face shield is exhausting, it's uncomfortable, it's hot, it's sweaty. I need a break to go and have some water, I can't do that on the ward, I've actually got to step off the ward and do it, so the staff are tired, overworked, dehydrated, wearing a lot of hot PPE, we still have to keep the hospital a nice cozy temperature for our patients and its our staff who are worn out and done the hard yards” (P4)

Fear induced behavioural changes were noted by four (33%) participants, and the subsequent impact this had on the workforce, including increased levels of paranoia, panic, and unprofessional workplace behaviours (Table 3, Q5 P1, Q8 P11). The increased levels of fear were described as having an impact on the decision-making capability of staff, from ensuring the workforce was comfortable with the protective controls implemented by the health service to be able to attend work and provide care, and understanding the risks associated with their actions and seeking out expertise to assist in the decision-making processes (Table 3, Q6 P1, Q3 P3).

#### 3.2 Getting staff into the mindset of a pandemic

Developing a mindset to be able to respond to the pandemic was identified as a sub-theme stemming from fear within the workforce. Five (42%) of the participant responses acknowledged the need to increase the workforce’s level of knowledge and understanding of the elements of the pandemic response, including disease transmission, the use of PPE, and maintaining vigilant practices, as essential elements to reducing fear and getting staff in a pandemic mindset to be able to provide care in a safe way that protects themselves and their patients (Table 3, Q11 P4, Q12 P11, Q13 P9).

#### 3.3 Infection prevention confusion

A lack of Infection prevention knowledge and skills within the workforce was identified by four (33%) of participants as a significant subtheme. The lack of knowledge and skill was identified to be associated with the fundamental elements of an infection prevention program and included a lack of understanding of disease processes and transmission, the differences between airborne and droplet transmission, and the appropriate and safe use of personal protective equipment (PPE) (Table 3, Q11 P4, Q12 P11, Q13 P9). A lack of exposure to infectious diseases in the specialist areas of mental health and midwifery was identified by two participants as a rational for the workforce lack of knowledge, both participants acknowledged a lack of familiarity and experience working with infectious diseases including the use of PPE (Table 3, Q 13 P9, Q16 P5). The transmission route of COVID-19 and the debate between aerosol and droplet transmission of COVID-19 was another barrier identified by several participants resulting in confusion and anxiety within the workforce (Table 3, Q20 P3, Q21 P2).

#### 3.4 Developing novel PPE programs

In response to the knowledge deficits within the workforce and the identified gaps in the education program eight (66%) of the participants acknowledged that the leadership group developed novel programs to facilitate the pandemic response and address the gaps. This included establishing a PPE sub-committee, a respiratory fit-testing program and re-developing the education programs to enable remote learning and introducing an online training component (Table 3, Q24 P11, Q27 P1, Q31 P5). Barriers to implementing this response were identified by two participants and included the rapid roll out of a P2 fit-testing program with limited access to resources (Table 1, Q30 P4), and the original PPE program was found to be lacking, and slow to address the concerns identified within the organisation and contributed to the confusion felt by the workforce (Table 3, Q25-26 P2).

#### 3.5 Adapting to change; implementing PPE processes within the workforce

The barriers associated with implementing programs to provide a safe work environment for the workforce and to facilitate changes was identified by six (50%) participants and included keeping up with rapid changes to PPE guidelines, implementing standardised practice across multiple facilities and embedding the changes within the workforce. PPE guidelines and recommendations changed frequently, resulting in confusion in the workforce, including the different terminology used of levels of PPE, and COVID levels of risk, and the requirements of the health service (Table 3, Q32 P11).

The participants identified that while the workforce were eventually able to adapt to the changing guidelines, it was not without its challenges. The workforce response to PPE use early in the pandemic was dramatic and a clear challenge for staff to when they were used to long lead times for change implementation, but over time they were able to demonstrate their ability to adapt to fast paced change (Table 3, Q33 P11, Q34 P5).

One participant identified the use of clear and reproducible guidelines as a key strategy to establish PPE compliance within the clinical environment, as it facilitated repetition and embedded practices within departments (Table 3, Q35 P3). The uncomfortable nature of wearing PPE for extended periods was identified as a barrier to PPE compliance by three participants, who noted that unless the workforce was used to wearing PPE before the pandemic, for example operating theatre staff, the workforce struggled to adopt the extended use of PPE (Table 3, Q38 P6, Q39 P11, Q40 P4).

##### Cross-sectional survey response

Despite the lack of infection prevention knowledge and skill being identified as a significant subtheme, when surveyed the front-line healthcare workers responded positively and indicated that they felt there were enough resources available, there were tools available to guide them when needed, and they felt confident in the use of the resources (Table 4). Staff indicated that on average they were confident in the fit of their N95 mask, 7.38 (SD 2.53), the PPE they were provided with would adequately protect them against COVID-19, 7.03 (SD 2.29) and that they understood the importance of the sequence of donning PPE, 8.75 (SD 1.79), and doffing PPE, 9.34 (SD 1.46). One possible explanation for this response lies in the implementation of the novel programs, education packages and frequent updates to guidelines, by the organisation leaders to address the identified deficits.

**Table 4:** Frontline healthcare workers perception of PPE preparedness during the pandemic

	**Strongly agree**	**Agree**	**Somewhat agree**	**Neither agree or disagree**	**Somewhat disagree**	**Disagree**	**Strongly disagree**
The recommended PPE are readily available in my department whenever they are needed	37.02% (184)	38.23% (190)	14.49% (72)	3.02% (15)	3.82% (19)	1.81% (9)	1.61% (8)
There is enough PPE supply for all healthcare staff in my department	31.48% (147)	43.47% (203)	13.28% (62)	3.64% (17)	4.50% (21)	2.36% (11)	1.28% (6)
I have had sufficient training in the correct use of PPE	53.36% (254)		34.87% (166)	6.93% (33)	3.99% (19)		0.84% (4)
I have a clear understanding of the indications for use of different types of PPE	59.17% (254)		32.53% (149)	6.93% (33)	3.99% (19)		0.84% (4)
There are sufficient visual reminders to remind on the use of PPE	64.47% (294)		27.63% (126)	5.26% (24)	1.75% (8)		0.88% (4)
The visual reminders on the following of PPE are a useful reminder to me	60.18% (269)		28.86% (129)	7.83% (35)	2.01% (9)		1.12% (5)

### Theme 4 PPE supply and demand

The fourth major theme identified was personal protective equipment (PPE) supply and demand. Subthemes within this major theme included (4.1) the crisis stage, (4.2) logistic challenges, and (4.3) centralised supply.

#### T4.1 The crisis stage

The majority of participants (8, 67%) reported that in the initial phase of the pandemic PPE supply and demand created a heightened level of anxiety and fear within the leadership group about ensuring there were adequate supplies of PPE to the workforce. However, three (25%) participants noted that while there was increased levels of anxiety at no point were they ever in a position to not be able to supply PPE to the workforce (Table 5, Q1 P4, Q3 P5, Q6 P6).

**Table 5:** Theme 4 PPE supply and demand

Themes	Subthemes	Organisational leaders
**4. PPE supply and demand**	The crisis stage	**Q1** “We’re faced with this crisis, that are we going to have enough set of gloves for a day in our ED are we going to be able to do this” (P4)
		**Q2** “You know masks were scarce at one point, they do chop and change the supply as well” [….] “And there were a few laughs along the way, trying on boiler suits you know” (P5)
		**Q3** “I think we've been reasonably okay with supply most of the time, but certainly in the beginning, obviously it was a little bit scary. I don't think we ever got to a point where we had to say to people, you know you can't have anything” (P5)
		**Q4** “I think the organisation was probably a little bit behind the eight ball, in the sense that a lot of other health services forecast potential shortfalls and accessed PPE prior to a centralized stockpile mechanism” (P3)
		**Q5** “I think the only time, on no it might have been twice, that I thought what on earth am I doing here, when I didn't I didn't think I had enough PPE to send my junior staff into ICU to look after COVID positive patients, but that was when what was coming out of Italy at that time was a lot of doctors dying” (P8)
		**Q6** “In the early days we were worried about supply, we had to really be careful about how we used our supply and be responsible, but again being responsible when people needed it they needed it, so there was no point where we were not able to have our staff in the PPE that they needed to be in” (P6)
		**Q7** “So, going from say whether it's a hand sanitizer or PPE we really struggled in the early stage to work out what our requirements are going to be and how we are going to cater that requirement” [….] “We just couldn't visualize demand because in the traditional days we work out what we need based on historical usage, so we can work out the numbers, but in the early stage we really struggled” (P7)
		**Q8** “My team in purchasing, we really struggled in the early days, because you know what the requirement is and you know you're not going to get that requirement. But some of the time you have to put a smile on your face, and face the business and say oh we're working on it we're going to get it, but in the back of your mind you know that there is nothing there that you can do it, so that first five to six months was a real struggle” (P7)
		**Q9** “At one stage we were looking at N95’s, within 6 or 12 months we used 25 years’ worth of N95. That’s the scale we were looking at on a normal day” (P7)
		**Q10** “And we were concerned partly about sort of our supply of N95 and at times gowns, but we never got to the stage where people were never supplied with the appropriate PPE” [….] “We got to a stage about discussing options for recycling N95s and gowns, and we get I think a fairly regular view of the stock take during that time” [….]“Although we had times where we thought okay we've only got 100 (gowns) left for the week what are we going to do” [….] “I think at the end we never really got to the situation where we couldn't get any PPE supplies, but there were certainly anxieties” (P10)
		**Q11** “We initially had people such as the OR nurse manager, ward managers, ringing up their usual suppliers as private phone calls and attempting to get products, so we could end up with a situation where different ward areas and different theatre areas had a diversity of equipment available” (P12)
		**Q12** “It left the leaders in a difficult position, because we had to explain why we weren't getting more (PPE), why we were making the decisions we were, trying to provide reassurance that that the information we were providing was based on best practice, based on trying to balance demand was in agreeance with guidance coming out of the department, so that that period was very challenging because obviously from an individual staff member perspective, there was a lot of differences of opinion from individual staff as to why some of the decisions were being made. That was a very challenging time because we're obviously trying to make what we thought were the most guideline concordant decisions that we thought were in the best interests of staff, but obviously when you're dealing with a crisis and demand is outstripping supply and you have to make difficult choices and yeah it was a very challenging time” (P3)
		**Q13** “The supply of the P2 masks that we were getting really early on didn't fit that brilliantly, they were built for an Anglo-Saxon male in a manufacturing environment, and we are an 85% female organisation from 150 different countries, so we had some real challenges in getting masks that fit and masks that were comfortable” [….] “And everybody saw you know the (news) footage of people will big bruising around their face” (P4)
		**Q14** “Early on I was monitoring incident reports about what people are reporting for their masks and there would be five to 10 a month about the issues people were experiencing, I'm not getting anything now, having said that, I know that you put a P2 mask on and someone says you're going to wear that for your 8, 10 or 12 h shift, exhausting” (P4)
	Logistics	**Q15** “Okay we need a whole lot of masks, right where do we get them from, where do we store them, who distributes them, who monitors stock levels. It's very easy in your original plan to say we would just get PPE and we would store it here, and you haven't necessarily thought through the volumes, or that supply and logistics element of it” (P3)
		**Q16** “We managed to get some space for the back-up stock, but we never anticipated that we will be needing to hold about 40,000 gowns as an emergency backup” [….] “But again, the human nature when the pandemic is over everyone will get on with life and forget about it until the next one, but this is something we need to work really closely with the business and see how we can cater for such an emergency situation” (P7)
		**Q17** “You see it was all pretty chaotic at that beginning period because of the supply chain issues generally, and a lack of overall supply” (P3)
		**Q18** “I actually think that there was actually good support between the sites, and I think there was understanding that one site was undergoing a lot more activity than the other at that time, and you know they could redeploy PPE as required” (P10)
	Centralised supply	**Q19** “The DHHS jumped on that wagon at the right time, as with any transition we struggled and they struggled, but they took control of the entire supply so there was no health service that can go outside of that network, and buy from the supplier, so they cut down that supply and brought it into a central supply chain” (P7)
		**Q20** “A N95 is the classic example, so we had three—four different brands and sizes of N95 available across the board, but when we go into that centralized supply chain network, only few products were available, and staff want to continue to use that product that they used to use, and we couldn’t cater that product” [….] “So in any transition it doesn't go perfect, but I’m really, really happy with the way the State Government responded, and the way they set up the network quickly and put a system in place and I’m confidently saying that they never let us down in any given day. We asked for say 10,000 gowns, and they probably gave us half of it, that's the case in the early days, but they never let a single day that they didn't give us anything” (P7)
		**Q21** “The DHHS are looking at the Health Share Victoria model, I think it is in place in NSW, so that every public hospital has to buy from that network, they centralise the product, they centralise and consolidate the brand etc., So I think down the track Victoria will adopt that model, that means you only get what they supply. Now they've got Health Share Victoria, but in the beginning everyone was scrambling for procurement and consumables and New South Wales was as well, but it got centralized a lot quicker And then that gave the organisation's confidence that that wasn't going to be an ongoing issue” (P7)
		**Q22** “with the lack of a centralised supply system the organisation was used to purchasing all of its own supplies often from private suppliers rather than a state supply centre, and one of the significant changes has been the centralisation of state supply, and notification of supply levels and supply chain reinforcement, which in the early days at least 6–9 months, was not very sound” (P2)
		**Q23** “I can remember, so the State supply chain they supply for free, but towards the end of the year they give us a report on what they suppled and how much its worth, and the report went to the CFO and he fell off the chair. We are talking about some $2.4 million dollars’ worth of PPE consumed across both hospital in that 12-month period. Which we never accounted for, and never predicted for, and it's just the way it is. And it is like the $2.4 million is just gone, you can get the physical stock but you can’t just articulate back to the business and say we've got that 40 or 50 pellets of gowns, but they were just gone within two days across both hospitals and aged care” (P7)

Two (12%) participants noted that the Organisation was behind in the preparation phase and forecasting potential PPE shortfalls compared to other health services and struggled to predict the PPE supply needed (Table 5, Q4 P3, Q7 P7). Two (12%) participants also noted the impact PPE supply and demand had on the workforce and the additional challenges that created with department managers attempting to access their own supply channels and communicating the PPE decisions based on supply and demand to the workforce (Table 5, Q11 P2, Q12 P3).

#### 4.2 Logistic challenges

The logistic challenges associated with monitoring stock levels and controls for PPE, along with recognising the sheer volume of PPE required were identified by three (25%) participants during the different phases of the pandemic. During the pandemic planning and preparation phase two (12%) participants noted that there was a lack of preparedness in identifying controls for PPE management as an issue, including storage of substantial volumes of PPE and forecasting reserve supplies (Table 5, Q15 p3, Q16 P7).

#### 4.3 Centralised supply

Two (17%) participants identified that the Victorian Department of Health centralisation of a state supply chain provided the health service with confidence that PPE supply would be maintained and would be equitable to all health services (Table 5, Q19 P7). One (8%) participant acknowledged that the transition to the centralised supply model was not without its challenges initially, largely relating to supply of specific products (Table 5, Q20 P7). While this issue created anxiety and fear within the leaders, at the time of survey the responses indicated that frontline staff did not feel this impact, and that the recommended PPE was readily available to them (strongly agree 37.02%, agree 38.23%) and that there was enough PPE available for all staff within their department (strongly agree 31.48%, agree 43.47%). The survey participants also indicated that they were confident with the use of PPE including donning and doffing and they felt protected by the PPE they were supplied with.

### Theme 5 Maintaining a workforce

The fifth major theme identified was around maintaining a workforce and continuing to provide care as an operational health service during a pandemic. Subthemes within this major theme were (5.1) the movement of people, and (5.2) the burden of contact tracing and staff furlough.

#### 5.1 The movement of people

The movement of people within a health service was identified by five (42%) participants as a significant challenge during all phases of the pandemic. In the planning and preparation phase identifying how to restrict the movement of people was a particular challenge, particularly in a tertiary hospital that required meetings, education sessions and staff groups that were required to move throughout the hospital (Table 6, Q1-2 P2, Q3 P10).

**Table 6:** Theme 5 Maintaining and normalising a workforce

Themes	Subthemes	Organisational leaders
**5. Maintaining and normalising a workforce**	Movement of staff and limiting in-person contact	**Q1** “Things like orientation where we have a dozen surgical JMOs appear on a day of change over, not having them all in the same room at the same time in face-to-face orientation, so we have had to implement the workflow and team structures around that. Things like the face-to-face education components, orientation components, hand over sessions, other meetings, other clinical meetings, basically all had to be done remotely” (P2)
		**Q2** “In a teaching hospital environment, it is not just about having meetings, but face-to-face education, engaging with the community, patient education sessions, effectively largely all cancelled” (P2)
		**Q3** “We're very susceptible because it's quite normal for medical staff to move across the areas of the hospital. Trying to restrict the movement of doctors was very critical, we were trying to work through models of maintaining doctors in particular areas, which then had its own sort of inefficiencies. There were additional handoffs (between teams), which were risky and it meant that there were longer length of stays and inefficiencies, and a loss of continuity of care for the patients” (P10)
		**Q4** “People didn't get it, so no social distancing, people were gathering without masks in the cafeteria, in the coffee area, their tea rooms. People would be in full PPE in any COVID areas and they go into a tea room and take it off and have a chat to their colleagues, because they all had to go for break, at the same time” [….] “Now we put the barriers in very early, how many people can be in a room, don't go and take your mask off, outside marquees for people to take breaks, and that we wanted people to sign in which they didn't, and still is an issue with tracking people and where they are” (P1)
		**Q5** “We had issues with the doctors always having to move in groups. All doctors would be in emergency at the same time, and so the risk is furloughing the whole entire medical group, similar in some respects to all the nurses having to go off on breaks at the same time and breaking those sorts of long-standing habits is incredibly challenging” [….] “Last year we believe that transmission also occurred because the ICU liaison nurses who had moved from ICU down into the wards as they normally do, and transmission occurred” (P1)
		**Q6** “We had to implement zoom, you know this online platform, how does that work, and I remember the first few meetings having to be on the phone to someone to walk them through how to have a face-to-face meeting” (P4)
		**Q7** “One of the key things I made a personal decision on was to go onsite every day, I could have said I’m an exec I can stay home” [….] “you actually have to have people on the ground, you have to have a presence and you have to have leadership. From my point of view because the COO and the program directors early on were across both sites, some days I would be the only high-level person on site. They can’t think oh everybody has abandoned them and everybody is just working from home, and that meant somebody was actually there to help escalate things and get it done for them and be like you guys just go and do your job care for the patients and we will sort out the rest” (P11)
	Burden of contact tracing	**Q8** “Certainly there were lots of community cases, but the transmission in ICU and on the fourth floor was tearoom driven” (P1)
		**Q9** “I've seen people getting quite antsy when they don't agree with a decision about a furlough or about you know what needs to happen, and I think we've just stuck together as a leadership group and you know the rules are the rules, and that is the decision and good luck with it” (P6)
		**Q10** “We had one incident, one of the first loads of furlough, and we lost quite a few theatre staff, and we were really concerned about our ability to continue, because obviously you can't run an obstetric unit without theatres. But we managed to get through that by cancelling all electives. And the theatres were only the skeleton staff we had left, just there for doing the caesareans, and we got through” (P6)
		**Q11** “All those furlough’s you think the world's falling apart around you, but the fortnight goes, and now it’s a week, and I think you learn to be more resilient as each one comes” [….] “And we did have an exposure in the nursery and I think we all held our breath for a little while, we didn’t get too many furloughed and we managed to work our way through it, and again I mean it is just sitting down and thinking logically about how you are going to manage this particular situation” (P6)
		**Q12** “This came on so quickly we just didn't have the staff, but that was recognized relatively early on and the organisation tried to support the infection control team as best they could. You have to have competent staff that's actually going to be helpful as well, you can throw 20 people at a department but if they actually can’t help it makes it difficult” (P11)
		**Q13** “We had people working really, really hard and intensely, but we had really poor systems, and then we had poor compliance with the systems” [….] “And at that stage I think we were still evolving our contact tracing processes and procedures” (P1)
	Furlough challenges	**Q14** “Now people are a lot more aware of it (furlough), I think people that did those big two weeks stints back in the early days, probably felt quite isolated and quite almost stigmatized from doing that. And now that sort of moved to well everyone almost got a turn, and I’ve missed out on my two weeks sitting at home” (P5)
		**Q15** “Everyone's just super tired you can see it walking around, I was talking to somebody about people going off and getting a COVID test because you can get paid COVID special leave, you do that and have a day off” (P5)
		**Q16** “We have a very low base of medical workforce, so availability of staff particularly in relation to furlough or sick leave was significant” [….] “We're still in a continual challenge at the moment, so it's not just maintaining your current level of staff we actually also have to get additional staff as well” (P10)

Two (17%) participants identified the existing practices of medical and nursing workforce groups was a risk in terms of large numbers of staff congregating together, moving throughout the hospital and also taking breaks together, was increasing the risk of furlough should transmission of COVID-19 occur (Table 6, Q3 P10, Q5 P1). One (8%) participant identified the implementation of changes to models of care as an attempt to restrict this behaviour, however this was not without its challenges, and had some implications for patient care including longer length of stays and additional handovers (Table 6, Q3 P10). With the implementation of work from home models for non-clinical staff, one (8%) leader identified the need for the leadership group to be present on-site and to provide the workforce with reassurance that they were supported (Table 6, Q7 P11).

#### 5.2 The burden of contact tracing and staff furlough

Five (42%) participants recognised the need for the organisation to have efficient systems in place and trained staff to support large scale contact tracing activities that lasted for an extended period (Table 6, Q12 P11, Q13 P1). Developing the resilience to deal with a reduced workforce due to furloughed staff was identified by one participant as having a burden on the health service (Table 6, Q10-11 P6). Maintaining a workforce due to furlough was identified by two (17%) participants as a challenge, partly due to the low base of medical workforce to begin with, and partly due to staff fatigue and increased awareness of COVID leave providing an excuse to have time off (Table 6, Q14-15 P5, Q16 P10).

## Discussion

The COVID-19 pandemic created unique situations that many healthcare leaders had not previously encountered. The broad nature of the pandemic response not only required leadership of an operational health service, but it also required leadership during a crisis with no clear end, and in roles that were newly defined to many. Leadership roles during a prolonged crisis are expected to be the commander and decision makers at the same time as being participative leaders that listen and build relationships between different groups to work collaboratively to solve the crisis [13].

Overall, the responses from the participants identified both strengths and barriers in the organisation’s response to the pandemic. Strong communication was identified as key, with a clear structure and the ability to get the leaders in the room together. The use of virtual tools and electronic meeting platforms, to facilitate this was an advantage. A study looking at the Victorian COVID-19 pandemic response across four health settings also identified clear and consistent communication as a key strategy that was vital to pandemic management [14]. Similarly, a study looking at healthcare workers well-being identified initiatives that provided open and inclusive communication from the leadership to the workforce would minimise fear and stressors that impact the workforces wellbeing [15] Between December 2021 and July 2022, 78 pandemic orders were made by the Victorian Minister for Health [16]. The frequent and fast changing guidelines provided additional challenges in communicating and implementing changes to the workforce. A lack of preparedness in pandemic planning was evident with the existing pandemic plan relating to influenza not fit for purpose for a novel respiratory virus. Previous pandemic events, like SARS and H1N1 influenza, have emphasised the importance of comprehensive pandemic management planning that is flexible, however hospitals often rely on generic, localised plans that are not designed for operationalising successful responses during protracted pandemics [17]. When a crisis strikes, single level organisations experience challenges and often find themselves ill prepared to meet the challenges of the uncertain and volatile environment [18]. During the pandemic the need for the command-and-control response was identified by the leaders, to make quick decisions bypassing the more traditional extended consultation process people were familiar with. However, the implementation of the incident command structure and the crisis management response at a scale not experienced by many of the key leaders had an impact on the outcome of the response. A similar study found that some healthcare workers and key personnel seized the opportunity created by the pandemic to promote and expand upon digital technologies, and were able to implement rapid infrastructure changes without the usual government and budgetary constraints [14].

Infection prevention and control practice standards for the management of COVID-19 were developed by federal and state governments and evolved rapidly as updated international experiences and advice were released. In the early stages of the COVID-19 pandemic, the availability of multiple guidelines had the potential to create confusion as to which guidelines should be followed at a local level. The communication systems within the various health agencies and government were viewed as fragmented, and at times it was unclear who was making decisions, which in turn enhanced the challenges in providing the workforce with the most relevant and recent information while avoiding oversaturation and overwhelming staff [14]. Due to the novel nature of the COVID-19 virus, information on transmission and recommendations for prevention evolved rapidly throughout the pandemic [19, 20]. The speed and frequency at which guidelines changed was also problematic and a considerable challenge that healthcare workers, organisational leaders, and infection prevention and control practitioners faced, particularly in the dissemination and implementation of the most up-to-date recommendations [10]. However, while the challenges in disseminating evolving information was experienced by the leaders, the survey participant responses indicated that frontline staff had confidence in the way the organisation communicated these changes During the crisis stage of the pandemic response, a lack of consistent PPE supplies was identified as one of the most significant issues impacting the leadership group, however the leaders interviews stated that they were able to maintain the supply of stock to their workforce.

### Limitations

There is a potential for bias in the responses from the front-line healthcare workers in the surveys, that cannot be validated through observation within the workplace, however the use of the survey was the most appropriate way to obtain the opinion of a large cohort of the workforce across the organisation. The large survey sample size does however provide confidence that the survey responses were representative of the views of clinicians working at the health service. This study was also only conducted at one organisation during the pandemic, therefore only obtaining the perspective of one group of leaders based on their experiences which could be influenced by existing practices and processes within the organisation. As participants were drawn from across different sites within the health service, this overcame potential bias associated with the differing impact of the COVID-19 pandemic at different study sites.

## Conclusion

The wide variety of challenges faced by the leaders during the pandemic identified several gaps within the organisations preparedness that were critical to the response. Health service organisations were required to respond rapidly and with some agility to meet the service needs of the organisation, requiring the implementation of a clear pandemic plan, with provisions for implementing a command structure, and embedded strategies to deliver clear communications, and to address workforce needs. The effectiveness of this hinges on preparedness and familiarity of these structures by key stakeholders, with the intention to provide the workforce with a controlled and coordinated response to alleviate anxieties and fear within the workforce, and to the community members it serves.

Future research looking at comparisons in the response between similar health care organisations could provide valuable insights into aspects of planning and preparedness to inform future responses.

## Data Availability

The datasets generated and analysed during the current study are not publicly available due to confidential nature of the participant data but are available from the corresponding author on reasonable request.

## Abbreviations

PPE: Personal Protective Equipment
COVID-19: Coronavirus disease
WHO: World Health Organization
QR code: Quick response code
HREC: Human Research Ethics Committee
NUMs: Nurse Unit Managers
CEO: Chief Executive officer
COO: Chief Operating Officer
ED: Emergency Department
DHHS: Department of Health and Human Services
